# Dehydration of methanol and ethanol over ferrierite originated layered zeolites – the role of acidity and porous structure†

**DOI:** 10.1039/d2ra00334a

**Published:** 2022-03-25

**Authors:** Aneta Święs, Andrzej Kowalczyk, Barbara Gil, Lucjan Chmielarz

**Affiliations:** Jagiellonian University in Kraków, Faculty of Chemistry Gronostajowa 2 30-387 Kraków Poland lucjan.chmielarz@uj.edu.pl

## Abstract

Ferrierites and their delaminated (ITQ-6) and silica intercalated (ITQ-36) forms, with the intended molar Si/Al ratios of zeolite layers of 30 and 50, were synthesized and tested as catalysts of methanol to dimethyl ether (DME) as well as ethanol to diethyl ether (DEE) and ethylene dehydration. It was shown that increased content of acid sites, especially of Brønsted type, resulted in more active catalysts of alcohol dehydration. Brønsted acid sites dominate in ferrierites and their delaminated forms (ITQ-6). Contribution of the Lewis type of acid sites increased in silica pillared ferrierites (ITQ-36) possibly by deposition of aluminium species on the surface of amorphous silica. Conversion of methanol to DME was not limited by internal diffusion of reactants in narrow pores of ferrierite. Such limitation was observed for synthesis of larger DEE molecules over ferrierites. The ITQ-6 catalysts with the opened interlayer structure presented better efficiency in ethanol to DEE conversion due to overcoming these diffusional restrictions. Moreover, selectivity to DEE over ITQ-6 was higher than in the presence of three-dimensional ferrierite.

## Abstract 2

Opened porous structures of ITQ-6 and ITQ-36, are more effective in catalytic dehydration of ethanol to diethyl ether than microporous ferrierite. Surface acidity determines catalytic performance of the zeolite catalysts in alcohol dehydration.

## Graphical abstract

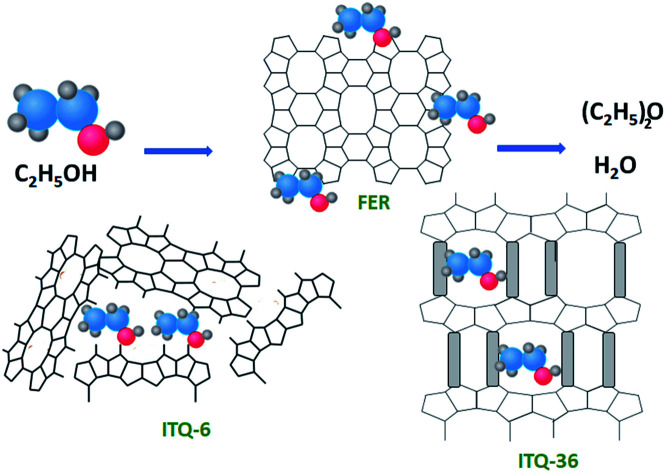

## Introduction

The reduction of exhaust emission in gasoline and diesel engines is one of the most important challenges. The introduction of appropriate additives to fuels can significantly reduce exhaust emissions, but also improve the properties of such fuels. In recent decades, the growing interest in the use of renewable and sustainable fuel additives has been observed. Alcohols, such as methanol and ethanol, as well as products of their dehydration, dimethyl ether and diethyl ether, belong to the most promising fuel additives.

Methanol is an attractive alternative of diesel fuel as well as fuel additive, meeting restrictive diesel particulate standards since methanol combustion forms very little soot. However, the temperature of methanol autoignition is high, which is an important disadvantage.^1^ This problem can be solved by replacement of methanol by dimethyl ether.

Dimethyl ether, DME, is considered as a promising, clean, and environmentally sustainable alternative fuel or fuel additive to diesel engines, owing to its high cetane number, low autoignition temperature as well as reduced emission of pollutants. Majority of the globally produced DME is blended with LPG,^2^ resulting in a fuel with significantly lower CO_2_ (by about 30–80%) and NO_*x*_ (by about 5–15%) emissions as compared with the LPG combustion.^3^ Moreover, since there is no C–C bond in the DME molecule, the formation of carbon nanoparticles during its combustion is effectively limited. DME is produced by two main technologies. The first one is based on direct conversion of syngas to DME (STD, syngas-to-dimethyl ether) over bifunctional catalysts active in syngas to methanol conversion as well as methanol to DME dehydration.^4,5^ The second technology is split into two separated processes. In the first step syngas is converted to methanol and then, after purification, methanol is dehydrated to DME in another reactor (MTD, methanol-to-dimethyl ether).^6,7^ The MTD process is effectively catalysed by acidic solid catalysts, such as γ-Al_2_O_3_,^8^ zeolites^9^ or modified clay minerals.^10,11^

Another, important fuel additive is ethanol. However, the use of ethanol in diesel engines is not fully satisfactory due to its low cetane number, low flash point, low calorific value, high water solubility as well as high corrosivity.^12,13^ This problem can be significantly solved by converting ethanol to diethyl ether (DEE), which has a higher cetane number, higher energetic content, lower auto-ignition temperature, broader flammability limits, and higher diesel miscibility than ethanol.^14,15^ Moreover, it was reported that incorporating diethyl ether into diesel results in an improving fuel combustion properties and therefore significant reduction of harmful emissions.^14–18^ Dehydration of ethanol to DEE can be carried out in the presence of strong homogeneous acid catalysts, such as H_2_SO_4_ and H_3_PO_4_. The main disadvantages of this technology are difficulties in separating DEE from the catalyst and corrosion of the installation operating in strongly acidic conditions.^18^ These problems can be significantly reduced by replacing of liquid solution of mineral acids for solid acidic catalysts, such as zeolites^9,19^ or modified clay minerals.^10,11^

As it was already mentioned, dehydration of methanol and ethanol to ethers, needs acidic catalysts. Zeolites belong to the group of the most promising catalysts of these reactions.^20–22^ Masih *et al.*^20^ reported very promising catalytic activity of small-pore zeolites, such as Rho, KFI and SSZ-13, with medium-strong acidic properties in the low-temperature methanol dehydration (≤200 °C). The measured methanol conversion was on the level of thermodynamical limit and selectivity to DME was 100% in the studied temperature range. Hassanpour *et al.*^21^ compared catalytic performance of various commercially available zeolites in the reaction of methanol to DME dehydration. The best catalytic performance presented H-ZSM-5, which was much more active than H-mordenite and NH_4_-mordenite as well as other zeolites in Na-form. Catalytic activity of zeolites was related mainly to density and strength of acid sites. Chen *et al.*^22^ studied the effect of crystal size of SAPO-11 zeolite (1-dimensional 10-membered ring medium pore channel) on efficiency of methanol to DME dehydration. The alcohol conversion on nano-SAPO-11 (20–30 nm) was higher than on both micro-SAPO-11 (2 μm, spheroidal crystals) and γ-Al_2_O_3_. It was suggested that the superiority of the nanosized zeolite may derive from larger amount of Lewis acid sites and higher diffusion efficiency.

The example of zeolites application in ethanol dehydration are studies of desiccated and dealuminated ZSM-5,^23^ which showed that adjustment of the acid sites concentration and their strength the reaction can direct the methanol conversion to DME or ethylene. Recently, Kuterasiński *et al.*^14,24^ reported increased catalytic activity of hierarchical MFI- and faujasite type zeolites modified by sonochemically assisted desilication in ethanol to DEE conversion. It was shown that sonochemically assisted desilication procedure resulted in higher average pore diameters in respect to the samples prepared by using traditional alkaline treatment method. Thus, it seems that internal diffusion is important in overall reaction efficiency.

Recently, ferrierites are of great interest as promising catalysts of methanol to DME conversion.^25–28^ Catizzone *et al.*^25^ reported high efficiency of this reaction the presence of ferrierites (Si/Al = 11) with different crystal sized. It was shown that decrease in the zeolite crystal size resulted in their increased catalytic activity in methanol dehydration. Smaller crystallites of ferrierite were less prone to carbon deposit formation and therefore more catalytically stable under reaction conditions. Moreover, it was shown that nanosized ferrierite with the crystal size up to 100 nm can be prepared by an innovative seed/surfactant-induced crystallization method.^26^ The decrease of crystal size from about 10 mm to about 100 nm increased the rate of methanol to DME conversion. This effect was explained by reduction of intracrystalline mass transfer limitations that improve the acid site accessibility. Moreover, it was reported that, for ferrierite zeolites, both the amount and the initial deposition rate of coke formed during the reaction were reduced when water was co-fed with methanol. Miletto *et al.*^27^ studied Cu–ZnO–ZrO_2_/ferrierite catalysts for one-pot CO_2_-to-DME conversion. Ferrierite play a role of acidic component of the hybrid catalyst for methanol to DME conversion. It was shown that bare ferrierite contains both Brønsted and Lewis acid sites, however Brønsted sites are mainly present in zeolites with the lower aluminium content. Moreover, it was suggested that acidity is strongly reduced after metals deposition due to possible exchange of protons for copper cations in some Brønsted acid sites resulting in Lewis. Such transformation of Brønsted to Lewis acid sites decreased catalytic activity of ferrierites in methanol to DME conversion. Thus, it could be postulated that Brønsted acid sites are more catalytically active in this reaction comparing to Lewis sites. On the other side, Prasad *et al.*,^28^ who studied Cu–ZnO–Al_2_O_3_/zeolite catalytic systems for direct synthesis of DME from syngas show superior activity of bifunctional catalysts containing ferrierite than other zeolites (ZSM-5, NaY or HY) as the solid acid component.

As it was postulated by many authors small-pore zeolites, including ferrierites, belong to the group of very promising catalysts of methanol to DME conversion.^20,22,25–28^ On the other hand, it was shown that nanosized zeolites, including ferrierites, are much more catalytically active than larger-sized ones.^22,25,26^ This effect was attributed to diffusional limitation. These reports were inspiration for the presented studies of delaminated (ITQ-6) and silica intercalated (ITQ-36) ferrierite based catalysts of methanol and ethanol dehydration to DME and DEE, respectively. ITQ-6 and ITQ-36 are characterized by opened interlayer structure and therefore improved internal diffusion of reactants. Different size of reactants, methanol and ethanol, as well as products of their dehydration, DME and DEE, is another criterium for evaluation of diffusion role in the overall efficiency of both processes. Moreover, the role of surface acidity of the zeolitic catalysts in alcohols dehydration is analysed and discussed.

## Experimental

### Synthesis of PREFER and FER

Synthesis of ferrierite precursor, PREFER, was based on the procedure reported by Corma *et al.*^29^ To prepare the reactant gel, fumed silica Aerosil 200 (Evonik Industries AG, Essen, Germany) used as silicon source, hydroxy(oxo)alumane Catapal B (Sasol, Johannesburg, South Africa) used as aluminum source, 4-amino-2,2,6,6-tetramethylpiperidine (R, Fluka, Germany) used as a structure directing agent, as well as NH_4_F (98%, Sigma-Aldrich, St. Louis, MO, USA), HF (49.8%, Sigma-Aldrich, St. Louis, MO, USA) and distilled water, were mixed in the molar ratios of 1SiO_2_ : *x*Al_2_O_3_ : 1R : 1.5NH_4_F : 0.5HF : 10H_2_O, where *x* = 0.015 and 0.01, to obtain the Si/Al molar ratios of 30 and 50, respectively. The obtained mixture was transferred to Teflon-lined stainless-steel autoclaves and stirred at 135 °C for 3 days. The resulting solid product was washed with distilled water, filtered, and dried at 60 °C overnight. The obtained products are denoted PREFER_30 and PREFER_50 for the materials with the Si/Al molar ratios of 30 and 50, respectively.

PREFER_30 and PREFER_50 were calcined at 600 °C for 6 h in air atmosphere to obtain three dimensional (3D) ferrierite zeolites, FER_30 and FER_50, respectively. Such thermal treatment resulted in condensation of the zeolite layers and formation three dimensional (3D) ferrierite zeolite structure.

### Swelling of PREFER

Swollen PREFER_30 and PREFER_50 materials were produced by dispersion of the lamellar ferrierite precursor (10 g) in a mixture containing: 40 g of distilled water, 200 g of cetyltrimethylammonium hydroxide solution (25 wt%, 50% exchanged Br^−^/OH^−^) and 60 g of tetrapropylammonium hydroxide (40 wt%, 70% exchanged Br^−^/OH^−^) with the final pH ≥ 12. The obtained slurry was stirred under reflux at 95 °C for 16 h.

### Synthesis of ITQ-6

The swollen PREFER materials were used for the synthesis of delaminated ITQ-6 zeolite. The slurry of swollen PREFER was treated with ultrasounds for 1 h (50 W, 40 kHz) and afterwards the pH of the mixture was decreased to 3 using concentrated hydrochloric acid (high purity grade, Honeywell, Charlotte, NC, USA). The solid product was separated by centrifugation (12 000 rpm, 20 min) and then washed with distilled water. Finally, the obtained samples were calcined at 600 °C for 6 h. Deaminated ITQ-6 zeolites with the intended Si/Al molar ratios of 30 (ITQ-6_30) and 50 (ITQ-6_50) were synthesized.

### Synthesis of ITQ-36

Swollen PREFER sample was mixed with tetraethyl orthosilicate (TEOS, 99%, Merck KGaA, Darmstadt, Germany) with the weight ratio of swollen PREFER to TEOS of 1 : 5. The obtained mixture was stirred at 90 °C for 16 h in nitrogen atmosphere. The obtained solid product was separated by filtration, washed with ethanol, and dried at 100 °C overnight. The second step was hydrolyzation of the modified zeolite by its dispersion in distilled water with the weight zeolite:water ratio of 1 : 10. This operation was conducted at 90 °C for 10 h. The final synthesis steps were washing with distilled water, drying overnight at 60 °C and calcination at 600 °C for 6 h. The ITQ-36 zeolites with the intended Si/Al molar ratios in the zeolite layers of 30 and 50 are denoted as ITQ-36_30 and ITQ-36_50, respectively.

Detailed procedures of ferrites synthesis as well as their delamination and intercalation are presented in our previous paper.^30^

### Characterization of catalysts

The structure of the zeolitic materials was analysed by X-ray diffraction (XRD) method. The XRD patterns of the zeolite samples were collected with D2 PHASER powder diffractometer (Bruker, Billerica, MA, USA). The diffractograms were taken in the 2*θ* range of 2–40 with a step of 0.02° and a counting time of 1 s per step.

The textural parameters of the samples were measured by nitrogen adsorption–desorption at −196 °C using 3Flex (Micrometrics, Norcross, GA, USA) instrument. Before the analysis, the zeolitic samples were outgassed under vacuum at 350 °C for 24 h. BET (Braunauer–Emmett–Teller) model was used for determination of the specific surface area. Micropore size distribution was estimated using the Horvath–Kawazoe model, while mesopore volume and mesopore area were determined using the BJH (Barrett–Joyner–Halenda) model. The *t*-plot method was applied for the micropore volume (at *p*/*p*_0_ = 0.98) and specific surface area of the micropores determination.

The chemical composition of the zeolitic samples was determined by using inductively coupled plasma optical emission spectroscopy method - ICP-OES (iCAP 7400, Thermo Scientific, Waltham, MA, USA). The zeolitic samples were dissolved under microwave radiation (Ethos Easy system, Milestone, Sorisole, Italy) in a mixture of hydrofluoric acid (high purity grade, Honeywell, Charlotte, NC, USA), hydrochloric acid (high purity grade, Honeywell, Charlotte, NC, USA) and nitric acid (high purity grade, Honeywell, Charlotte, NC, USA).

IR spectra were measured using Tensor 27 (Bruker, Ettlingen, Germany) spectrometer equipped with MTC detector, at spectral resolution 2 cm^−1^. Zeolites were pressed into self-supporting wafers with the density of *ca.* 8–10 mg cm^−2^ and activated *in situ* at 450 °C for 1 h at high vacuum (10^−5^ mBar) in a home-made quartz cell, equipped with CaF_2_ windows. Cell construction allowed *in situ* activation, measurement of the spectra at chosen temperature and adsorption of gases and vapours inside the infrared spectrometer. The concentration of acid sites was evaluated based on ammonia adsorption at 100 °C, using the absorption coefficient *ε*(LAS) = 0.022 cm^2^ μmol^−1^ (1620 cm^−1^ maximum) and *ε*(BAS) = 0.147 cm^2^ μmol^−1^ (1450 cm^−1^ maximum).^31,32^ In a typical experiment ammonia was adsorbed in excess (*ca.* 20 Torr equilibrium pressure) and then desorbed for 15 minutes at the adsorption temperature to remove gas phase and weakly adsorbed species. All spectra presented in this paper are recalculated to the same mass of the pellet (10 mg, the pellet surface 3.14 cm^2^).

### Catalytic tests

The zeolite samples were tested in the role of the catalysts for methanol and ethanol dehydration to dimethyl ether (DME) and diethyl ether (DEE), respectively. In all catalytic tests 100 mg zeolite was used. The catalyst sample was placed in flow fixed-bed quartz microreactor on the quartz wool plug and outgassed in a flow of pure helium at 500 °C for 30 min. The catalytic test was carried in the temperature range of 100–300 °C with a heating rate of 10°C min^−1^ using gas mixture containing an alcohol (3.9 vol% of methanol or 3.3 vol% of ethanol determined by their volatility at 0 °C, which was saturation temperature) diluted in helium with the total flow rate of 20 mL min^−1^. Concentrations of reactants were analysed by gas chromatograph (SRI 8610C) equipped with methanizer and FID detector. The operating temperature of chromatography column, depending on the reaction, was 120 °C for methanol dehydration or 180 °C for ethanol dehydration. For the most active sample, an additional isothermal stability test of dehydration of methanol was done at 225 °C for 60 h with the same composition and total flow of the reaction mixture as in polythermic catalytic tests.

## Results and discussion

**Fig. 1:**
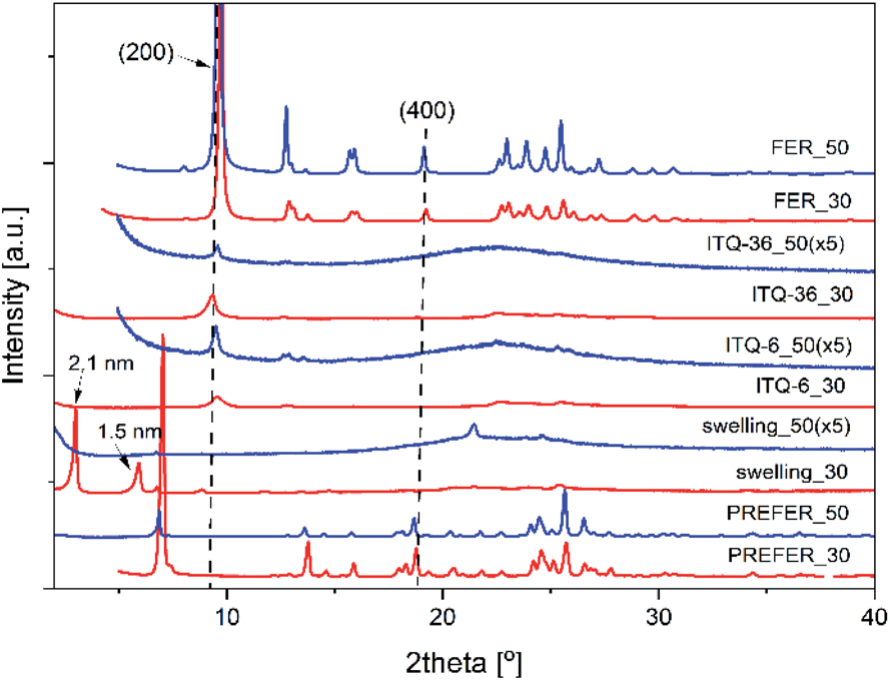
X-ray diffractograms of ferrierite precursors (PreFER), their swollen forms (swelling), ferrites (FER) as well as their delaminated (ITQ-6) and silica intercalated (ITQ-36) forms.

The structure of the zeolitic precursors and changes occurring in the subsequent steps of their modifications were monitored by X-ray diffraction method (Fig. 1). In diffractograms of the zeolite precursors, PREFER_30 and PREFER_50, the (200) diffraction peaks at 2*Θ* about 6.8–6.9°, characteristic of the interlayer distance of about 1.3 nm, were identified.^33^ The presence of the (400) diffraction peak, located at 2*Θ* about 18.6–18.7°, proves the layered structure of PREFER_30 and PREFER_50. The (200) and (400) diffraction peaks were shifted to 2*Θ* about 9.7° and 19.3°, respectively, after calcination of the zeolite precursors. This effect indicates the condensation of zeolite layers with the formation of three dimensional (3D) ferrierite structure (samples FER_30 and FER_50, Fig. 1).

Delamination and intercalation of the zeolite precursors, resulting in ITQ-6 and ITQ-36, need their swelling. As it can be seen (Fig. 1), the swelling procedure, which is conducted in a basic medium, in the case of PREFER_30 resulted in a shift of the (200) and (400) diffraction peaks into 2*Θ* about 3.0° and 11.8°, respectively, indicating an increase of the interlayer distance to about 2.1 nm. Decrease in the intensity of these reflections is attributed to partial disordering of the parallel orientation of the zeolite layers. This effect was much more significant for PREFER_50. In this case diffraction peaks related to the ordered zeolite layers disappeared indicating formation of delaminated structure, called the structure of “house of card”. Another important difference in diffractograms of swelled PERFER_30 and PREFER_50 is appearance of broad diffraction peak in the 2*Θ* range of 15–30° (Fig. 1), characteristic of amorphous silica^34^ in diffractogram of the high-silica sample (PREFER_50). As it was already mentioned, the swelling process was conducted under basic conditions, which resulted in a partial leaching of silicon from the zeolite layers. This effect was significantly more significant for the high-silica precursor. Possibly, in the next step amorphous silica aggregates were formed in solution and re-deposited on the external surface of the zeolite grains.

Textural parameters as well as the nature of acid sites and the Si/Al molar ratio in the samples are presented in Table 1. First, in ferrierites, FER_30 and FER_50, micropores dominate, however there is also a significant contribution of larger pores. Intercalation of layered zeolite with silica pillars, resulting in ITQ-36_30 and ITQ-36_50, as well as delamination of layered zeolite, resulting in ITQ-6_30 and ITQ-6_50, decreased micropore volume and increased mesopore volume. These effects indicate the opening of the interlayer space in zeolites and prove successful intercalation and delamination of zeolite precursors.

**Table 1:** Textual parameters, molar Si/Al ratios and distributions of surface acid sites in ferrierites and their delaminated (ITQ-6) and silica intercalated (ITQ-36) forms^a^

Sample	*S*_BET_ [m^2^ g^−1^]	*V*_micro_ [cm^3^ g^−1^]	*V*_meso_ [cm^3^ g^−1^]	Si/Al [mol mol^−1^]	BAS [μmol g^−1^]	LAS [μmol g^−1^]	BAS + LAS [μmol g^−1^]
FER_30	376	0.13	0.08	22	233	43	276
FER_50	377	0.13	0.10	64	47	0	47
ITQ-6_30	394	0.07	0.46	22	271	43	314
	(412)	(007)	(007)				
ITQ-6_50	372	0.07	1.12	214	15	14	39
ITQ-36_30	345	0.08	0.29	23	211	71	382
ITQ-36_50	265	0.02	0.82	224	2	114	116

a *S*_BET_ – specific surface area determined using BET model; *V*_micro_ – micropore volume; *V*_meso_ – mesopore volume; BAS – Brønsted acid sites; LAS – Lewis acid sites; values in brackets are related to the catalysts after 3 catalytic cycles.

The Si/Al molar ratios of the zeolitic samples, determined by their chemical analyses, are slightly different from intended values (Table 1). For FER_30 the intended Si/Al molar ratio was 30, while the measured value is 22. In the case of FER_50, it was planned to have the Si/Al ratio of 50, while the measured value is 64. In the case of PREFER_30, delamination the Si/Al ratio is the same as in FER_30, while its intercalation with silica pillars resulting in ITQ-36_30 only slightly increased silica content. Significantly more complicated situation is observed for high silica samples. As it was shown by the result of XRD analysis, part of silica was leached from the zeolite layers under basic conditions of swelling and then amorphous silica precipitated in solution was re-deposited on the surface of zeolite grains. This effect was observed only for high-silica zeolite, which is possibly less stable under basic conditions of swelling process. In the next step of the ITQ-6_50 synthesis, such swollen PREFER_50 was treated in acidic conditions (pH = 3), what possibly resulted in effective leaching of aluminum from the partially degraded zeolite layers. Partial dealumination of PREFER_50 under swelling conditions, as process assisting silicon leaching, cannot be also excluded. Intercalation of PREFER_50 with silica pillars to obtain ITQ-36_50 also resulted in the increase of the Si/Al molar ratio, which was significantly higher comparing to ITQ-36_30.

The nature of acid sites in the zeolitic samples was studied by IR analysis of ammonia adsorbed species (Table 1). In the ferrierite samples, FER_30 and FER_50, Brønsted acid sites (BAS) dominate. In zeolite with higher aluminum content also Lewis acid sites (LAS) are present, while in high-silica ferrierite such sites were not found. Delamination of the samples with higher aluminium content (ITQ-6_30) only slightly modified contribution of BAS and LAS. For the sample intercalated with silica pillars, ITQ-36_30, a decrease in BAS and increase LAS contribution was observed (Table 1). Much more significant differences in the nature of acid sites were identified for delaminated high silica zeolite (ITQ-6_50). Comparison of FER_50 and ITQ-6_50 shows the formation of LAS and significant reduction in BAS contribution in delaminated zeolite. As it was already mentioned, in the case of ITQ-6_50 dealumination and deposition of amorphous silica on the zeolite grains were observed. Even more significant changes in contribution of acid sites were observed for the silica intercalated zeolite, ITQ-36_50, containing mainly LAS with only small contribution of BAS (Table 1). Thus, it seems that such significant changes in the sample composition are reflected also in the nature of acid sites. Analysis of the results presented in Table 1 shows that the presence of amorphous silica results in LAS. Pure amorphous silica should not exhibit any acidity, however it is possible that under basic and acidic treatment of the zeolite samples some aluminium species were deposited on such amorphous silica aggregates and play a role of LAS. Of course, this scientific hypothesis should be verified by the future studies.

**Fig. 2:**
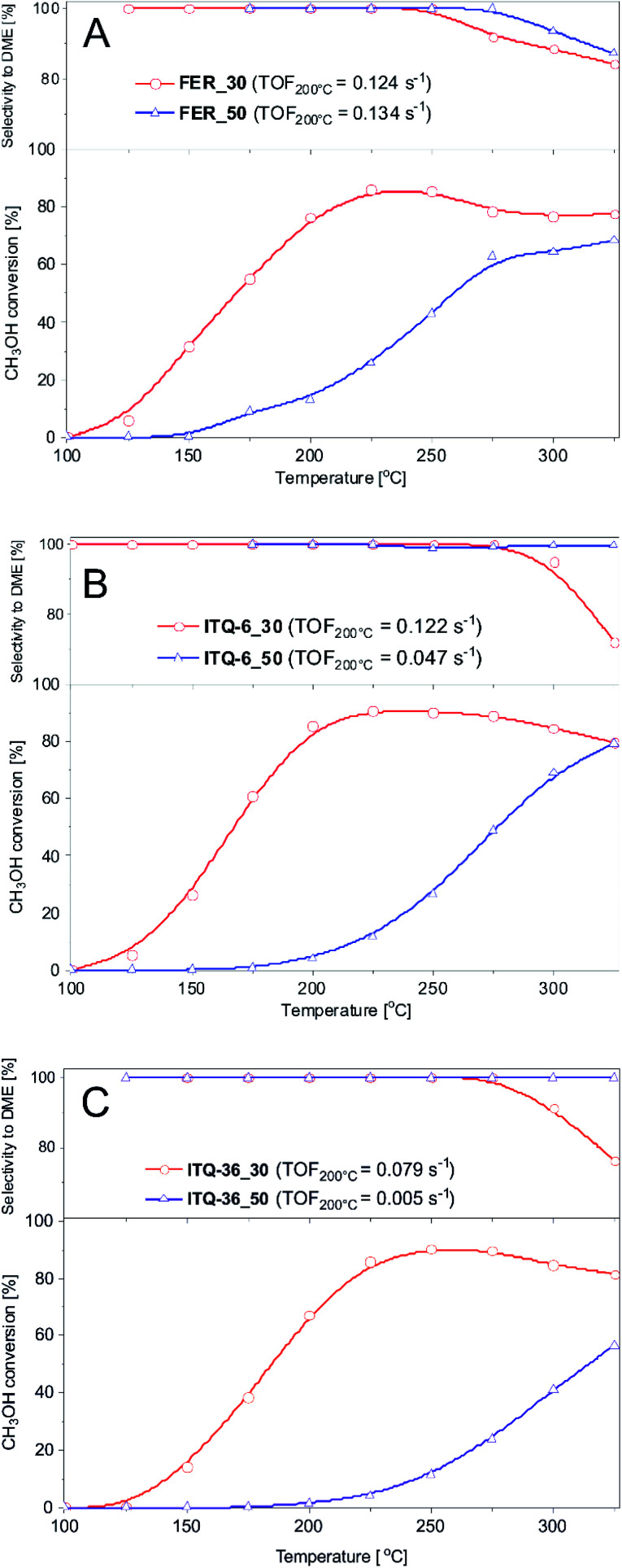
Results of methanol to DME dehydration over ferrierite (A), ITQ-6 (B) and ITQ-36 (C) catalysts. Reaction conditions: 3.9 vol% of methanol diluted in helium, flow rate of 20 mL min^−1^, catalyst – 100 mg.

Zeolitic samples were tested as catalysts of methanol to dimethyl ether (DME) and ethanol to diethyl ether (DEE) conversion. Results of methanol dehydration, presented in Fig. 2, show that the efficiency of this process depends on surface acidity of the catalysts as well as their porous structure. FER_30, ferrierite with the lower Si/Al ratio, and therefore higher acidity, presented much better catalytic activity comparing to FER_50 with the lower content of acid sites (Fig. 2A and Table 1). In the case of FER_30, the methanol to DME conversion started at about 100 °C and increased to the level of 86% at 225 °C. Decrease in the methanol conversion, observed at higher temperature, is related to thermodynamical restriction of this reaction.^35^

The methanol dehydration in the presence of FER_50 started at temperature higher by about 50 °C and was significantly lower than for FER_30 in the studied temperature range. DME and water were the only reaction products up to 225–250 °C, while at higher temperatures side products, such as formaldehyde, carbon monoxide and methane were also identified.

In the case of the ITQ-6 samples, the ferrierite zeolite layers form delaminated structure containing micropores in the zeolite layers and mesopores, which are interlayers spaces. Similarly to ferrierites, also in the case of ITQ-6, the zeolite sample with higher content of acid sites, ITQ-6_30, presented significant better catalytic performance in methanol to DME dehydration (Fig. 2B and Table 1). The methanol conversion started at about 100 °C and increased to the level of 92% at 225 °C. Higher catalytic activity of ITQ-6_30 than FER_30 could be related to slightly higher concentration of Brønsted acid sites (BAS) in the former catalyst (Table 1). Another possible explanation of this difference in catalytic performance could be opened delaminated structure of ITQ-6_30 with less restricted internal diffusion of reactants. Important advantage of the ITQ-6_30 catalyst is its broader range of the selective methanol to DME conversion in comparison to FER_30. The side reaction products in this case were observed at 275 °C, thus at temperature higher by at least 25 °C comparing to FER_30. This effect is possibly related to much easier evacuation of DME from larger pores of ITQ-6_30 than from micropores of FER_30 and therefore lower risk of the further DME transformation into by-products.

The ITQ-36_30 and ITQ-36_50 samples, are also characterized by open interlayer structure, which is stabilized by silica pillars located between ferrierite layers. Also in this case, the catalyst with the higher content of acid sites, ITQ-36_30, presented much better catalytic activity than intercalated zeolite with the lower surface acidity, ITQ-36_50 (Fig. 2C and Table 1). The methanol conversion in the presence of ITQ-36_30 started at about 100 °C and reached 91% at about 250 °C. Thus, the maximum of methanol conversion was located at temperature higher by about 25 °C in comparison to FER_30 and ITQ-6_30. Analysis of the LAS and BAS distribution in these samples shows that in ITQ-6_30 the contribution of BAS is lower and LAS is higher than in FER_30 and ITQ-6_30 (Table 1). Thus, the various contribution of LAS and BAS could be responsible for the shift of the methanol conversion maximum in the case of ITQ-36_30.

The turn-over-frequency (TOF) values calculated for the reaction of methanol to DME at 200 °C are presented in Fig. 2. In TOF calculations it was assumed that each acid site, determined by ammonia sorption (Table 1), plays a role of catalytically active site. The highest TOF values were determined for ferrierites (Fig. 2A), while a very significant decrease of this parameter was observed for ITQ-6_50 (Fig. 2B) as well as ITQ-36_50 and ITQ-36_30 (Fig. 2C). Comparison of TOF values with the contribution of BAS and LAS in the samples leads to the hypothesis that BAS are more catalytically active in the methanol conversion at 200 °C than LAS. Of course, this hypothesis should be verified by additional experiments.

**Fig. 3:**
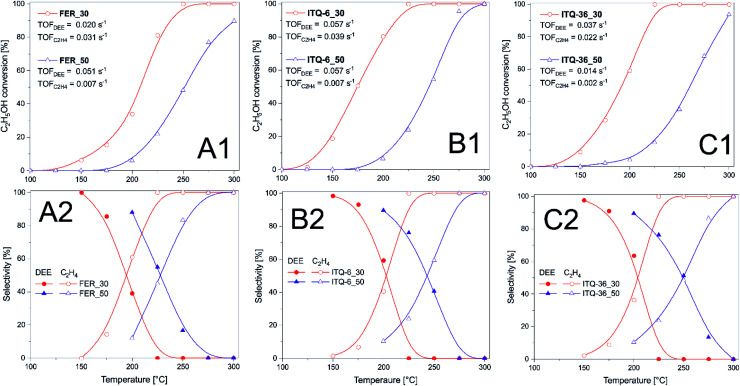
Results of ethanol to DEE dehydration over ferrierite (A), ITQ-6 (B) and ITQ-36 (C) catalyst. Reaction conditions: 3.3 vol% of ethanol diluted in helium, flow rate of 20 mL min^−1^, catalyst – 100 mg.

Zeolitic catalysts were also studied in dehydration of ethanol to diethyl ether (DEE) and ethylene (Fig. 3). Due to thermodynamic restrictions dehydration of ethanol to DEE is privileged at lower temperatures, while formation of ethylene dominates at higher temperatures.^36^ Similarly to dehydration of methanol, also in the case of ethanol dehydration the catalysts with the higher content of acid sites were found to be more catalytically active. Ethanol conversion in the presence of FER_30 started at about 100 °C and the level of 100% was obtained at about 250 °C. In the case of the catalyst with lower acidity, FER_50, reaction started at 150 °C and 90% of methanol conversion was obtained at 300 °C. Delamination and intercalation of ferrierite precursors resulted in more active ITQ-6_30 and ITQ-36_30 catalysts able to completely dehydrate ethanol at about 225 °C (Fig. 3). Important issue in ethanol dehydration is selectivity to the reaction products - DEE and ethylene. In the case of ferrierite catalysts, a decrease in selectivity to DEE and increase in selectivity to ethylene is observed at lower temperature than in analogous catalysts based on ITQ-6 and ITQ-36. Thus, delamination and intercalation of the ferrierite zeolite not only increased their catalytic activity in the reaction of ethylene dehydration but also shifted the formation of DEE into higher temperatures (Fig. 3). The turn-over-frequency (TOF) values calculated for the reaction of methanol to DEE and ethylene at 200 °C are presented in Fig. 3. Similarly, to TOF calculation for methanol conversion, also in these calculations it was assumed that each acid site, determined by ammonia sorption (Table 1), plays a role of catalytically active site. The highest TOF values related to ethanol to DEE (TOF_DEE_) as well as ethylene (TOF_C_2_H_4__) conversion were determined for ITQ-6_30 (Fig. 3A(1)). In the case of FER_30, the TOF_C_2_H_4__ value is higher than TOF_DEE_. While for these same reaction conditions the opposite effect, TOF_DEE_ higher than TOF_C_2_H_4__, was observed for ITQ-6_30 and ITQ-36_30 (Fig. 3B(1) and C(1)). It is possible that in small micropores of ferrierite the conversion of ethanol to small molecule of ethylene is privileged, while in larger pores of ITQ-6_30 and ITQ-36_30 the formation of larger molecules of DEE is more favourable. In the case of the catalysts with lower acidity, which were significantly less catalytically active, the TOF_DEE_ values are higher than TOF_C_2_H_4__ showing that the conversion of ethanol to DEE is more favourable over these catalysts at 200 °C.

**Fig. 4:**
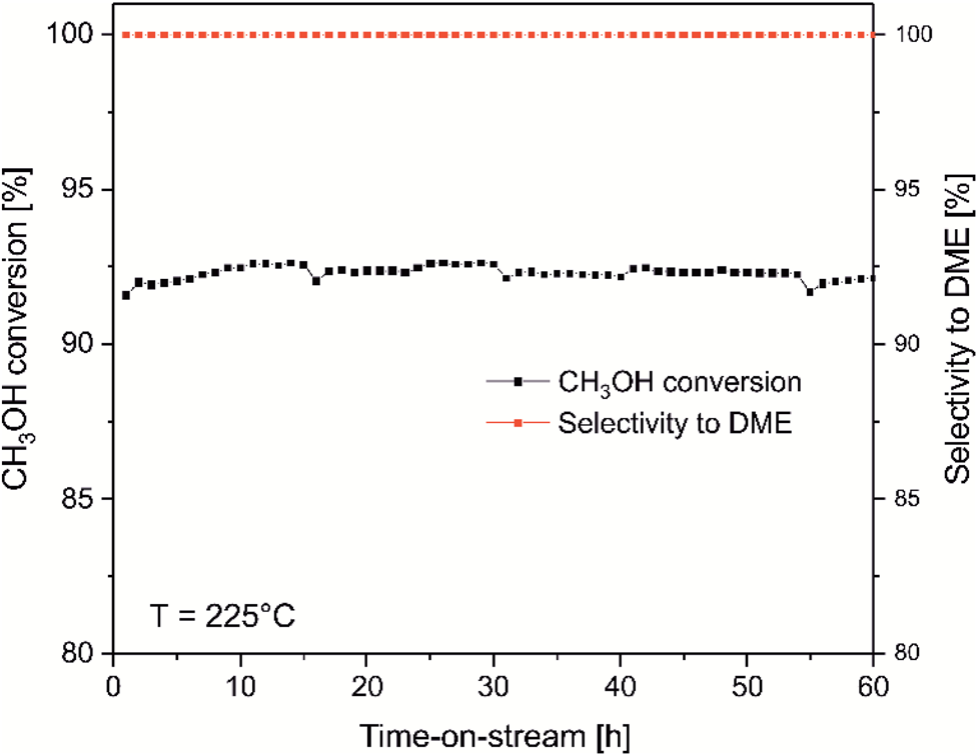
Results of isothermal methanol to DME dehydration over ITQ-6_30. Reaction conditions: 3.9 vol% of methanol diluted in helium, flow rate of 20 mL min^−1^, temperature – 225 °C, catalyst – 100 mg.

**Fig. 5:**
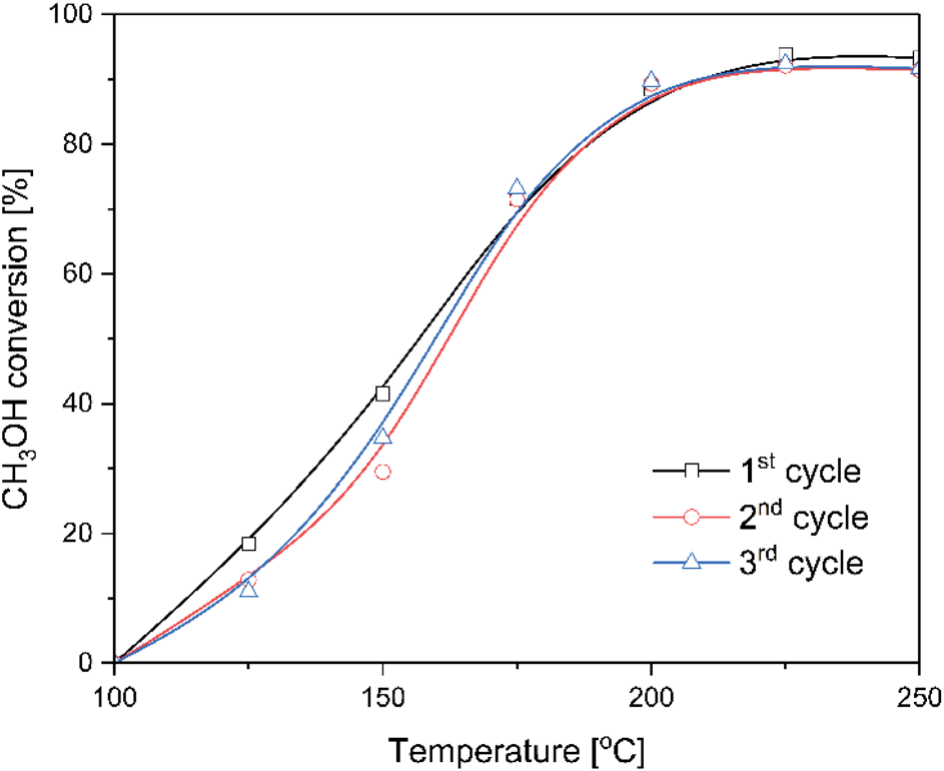
Subsequent catalytic runs for ITQ-6_30 catalyst in methanol to DME dehydration. Reaction conditions: 3.9 vol% of methanol diluted in helium, flow rate of 20 mL min^−1^, catalyst – 100 mg.

For the most active catalysts of methanol to DME conversion, ITQ-6_30, isothermal stability tests at 225 °C was done (Fig. 4). As it can be seen, the methanol conversion was in the range of 92–93% during 60 hours of the test. DME and water vapour were found to be the only reaction products. Thermogravimetric analysis of the spent catalysts showed no carbon deposit formation under reaction conditions (results not presented). Moreover, for the ITQ-6_30, three subsequent catalytic tests of methanol to DME conversion were done (Fig. 5). As can be seen the reproducibility of the catalytic results in each run is very good, indicating again high stability of the studied catalyst. The comparison of XRD diffractograms of fresh and used ITQ-6_30 catalyst shows no significant differences (ESI†), similarly to textural parameters of this sample before and after three catalytic runs (Table 1).

Marosz *et al.*^9^ studied MCM-22 zeolites and their delaminated and silica intercalated forms as catalysts of methanol and ethanol dehydration. In general, the conversion and selectivity profiles for these catalysts are similar to the catalytic systems presented in this work. Slightly higher activity of the MCM-22 originated catalysts is possibly related to the lower Si/Al ratio and therefore higher content of surface acid sites which play a role of active sites in alcohols dehydration. Moreover, ferrierite contains significant contribution of relatively small 8MR pores, which limits the internal diffusion rate of reactant. Majority of other reported in literature catalytic systems for methanol and ethanol dehydration, such as γ-Al_2_O_3_,^10^ intercalated clay minerals,^10,11^ alumina modified mesoporous silicas for SBA-15 type,^37^ presented significantly lower catalytic activity. Thus, zeolite-based catalysts seem to be very promising for methanol and ethanol dehydration.

**Fig. 6:**
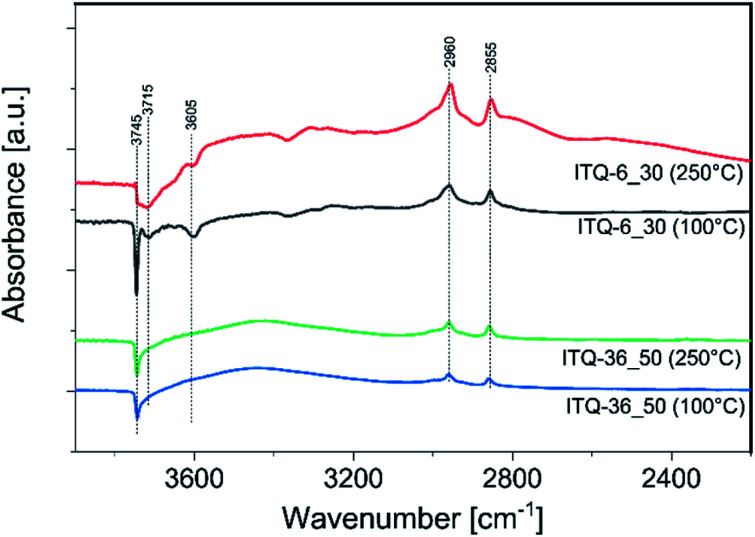
Differential IR spectra of ITQ-6_50 and ITQ-36_30 preabsorbed with methanol and treated at 100 and 250 °C.

To have more insight into interaction of methanol with the surface of zeolitic catalysts, the IR spectra for the samples preabsorbed with methanol molecules were recorded. Such measurements were done for the most catalytically active sample, ITQ-6_30, and the less active one, ITQ-36_50, treated with methanol and then heated to 100 and 250 °C. The spectra are presented in differential forms – the spectrum of outgassed sample was subtracted from the spectrum of the sample treated with methanol (Fig. 6). The negative band observed at 3745 cm^−1^ in a spectrum of ITQ-36_50 treated at 100 °C is related to the consumption of isolated silanol groups upon the sorption of methanol species.^38,39^ The positive bands at 2960 and 2855 cm^−1^ are related to adsorbed methoxy groups and indicate that methanol adsorbs and dissociates over this sample.^38,39^ An increase in the reaction temperature from 100 to 250 °C resulted in the methanol conversion enhancement from 0 to about 12% in the presence of the ITQ-36_50 catalyst (Fig. 2). On the other hand, intensity of negative band related to the consumption of isolated silanol groups by methanol species sorption as well as positive bands indicating the presence of methoxy groups are nearly the same for the catalyst treated at 100 and 250 °C (Fig. 6). Thus, it seems that methoxy species attached to isolated silanol groups present in ITQ-36_50 are not reactive in the DME synthesis or their reactivity in this process is very limited at temperatures below 250 °C. Sorption of methanol on the surface of ITQ-6_30, similarly to ITQ-36_50, resulted in the consumption of isolated silanol groups upon the sorption of methanol species (band at 3745 cm^−1^) and formation of the methoxy groups (bands at 2960 and 2855 cm^−1^).

The assignment of the small band at about 3715 cm^−1^ is not straight forward, but recently such band was suggested to be a result of different hydroxyl groups consumption by methoxy species.^40^ Moreover, in the spectra of the ITQ-36_50 sample additional negative band at 3605 cm^−1^, is attributed to consumption of Brønsted acidic sites, ≡Si–OH–Al≡, by methoxy species.^40^ The absence of the band related to hydroxyls associated with extra-framework alumina species at about 3650 cm^−1^ shows that aluminium is present nearly exclusively in the zeolite framework positions.^41^

An increase in reaction temperature from 100 to 250 °C resulted in the methanol conversion enhancement from 0 to over 90% in the presence of the ITQ-6_30 catalyst (Fig. 2). As can be seen in Fig. 6, in the spectrum recorded at 250 °C the intensity of negative bands assigned to the consumption of Brønsted acidic sites and isolated silanol groups by methoxy species was significantly reduced in comparison to the spectra obtained at 100 °C. Similarly, also intensity of the bands characteristic of methoxy groups at 2960 and 2855 cm^−1^, was significantly reduced after temperature increase to 250 °C. Thus, it seems that methoxy species formed on Brønsted acidic sites are transient species in DME production. Possibly, the reaction of methanol with isolated silanol groups also results in the formation of methoxy species, which in the next step are converted to DME. However, the difference in reactivity of methoxy groups anchoring at such isolated centres in the ITQ-36_50 and ITQ-6_30 samples is surprising. Comparison of these bands intensity for both catalysts may suggest that the content and therefore also surface density of methoxy species anchored on such isolated sites is much higher in the case of ITQ-6_30, which were much more reactive in DME production. Thus, the reaction between methoxy species anchored on such isolated sites, according to the Langmuir–Hinshelwood mechanism, cannot be fully excluded. Of course, this scientific hypothesis should be verified in the future studies.

## Conclusions

Ferrierites and their delaminated (ITQ-6) and silica intercalated (ITQ-36) were found to be active and selective catalysts of methanol to DME and ethanol to DEE conversions. Their catalytic activity in both processes depend on surface acidity as well as porous structure of the zeolitic samples. The samples with higher content of aluminium incorporated into zeolite framework, and therefore higher concentration of acid sites, presented much better catalytic performance than high-silica zeolites. Brønsted acid sites, which seem to be more active in alcohols dehydration, dominate in ferrites and their delaminated (ITQ-6) forms. Intercalation of zeolite precursors with silica pillars (ITQ-36) significantly increased contribution of Lewis acid sites, possibly by re-deposition of aluminium species from solution on the surface of amorphous silica. Delamination of ferrierite with higher alumina content (ITQ-6_30) did not significantly change reaction rate of methanol dehydration, expressed as turn-over-frequency (TOF), indicating leak of internal diffusion limitations of methanol and DME in narrow pores of ferrierite. The differences in the TOF values observed for the ITQ-36 samples as well as high-silica ITQ-6 are possibly related to the increased contributions of less active Lewis acid sites. Comparison of ethanol to DEE dehydration over ferrierite and ITQ-6 shows that TOF values are higher for delaminated zeolite, especially in the case of the sample with the higher aluminium content. Thus, it could be suggested that internal diffusion of DEE molecules in small micropores of ferrierites limits the over rate of ethanol to DEE conversion. Moreover, the rate of ethanol to DEE conversion, expressed as TOF_DEE_, increased after delamination of ferrierite with higher aluminium content nearly by three times. This effect is possibly attributed to the presence of larger pores in ITQ-6_30 with the improved internal diffusion of relatively large DEE molecules. This effect is also observed for silica intercalated ferrierite, ITQ-36, however interpretation of these results is more speculative due to significant changes in contribution of Brønsted and Lewis acid sites in this sample as well as their different reactivity in ethanol conversion.

## Author contributions

A. S.: methodology, investigation, data curation, writing—review & editing; A. K.: investigation; B. G.: investigation; L. C.: conceptualization, methodology, project administration, supervision, writing—original draft, writing—review & editing. All authors have read and agree to the published version of the manuscript.

## Conflicts of interest

There are no conflicts to declare.

## Supplementary Material

RA-012-D2RA00334A-s001
