# Supplementary material for: Dehydration of methanol and ethanol over ferrierite originated layered zeolites – the role of acidity and porous structure
Source: RSC Adv. 2022 Mar 25;12(15):9395–403. doi: 10.1039/d2ra00334a (PMC8985092; doi:10.1039/d2ra00334a)
Supplement: RA-012-D2RA00334A-s001 [file RA-012-D2RA00334A-s001.pdf]

Electronic Supplementary Material (ESI) for RSC Advances.
This journal is © The Royal Society of Chemistry 2022

Dehydration of methanol and ethanol over ferrierite originated layered zeolites – the role of acidity and porous structure

Aneta Święs,[a] Andrzej Kowalczyk, [a] Barbara Gil [a] and Lucjan Chmielarz* [a]

[a.] *Jagiellonian University in Kraków, Faculty of Chemistry, Gronostajowa 2, 30-387 Kraków, Poland*

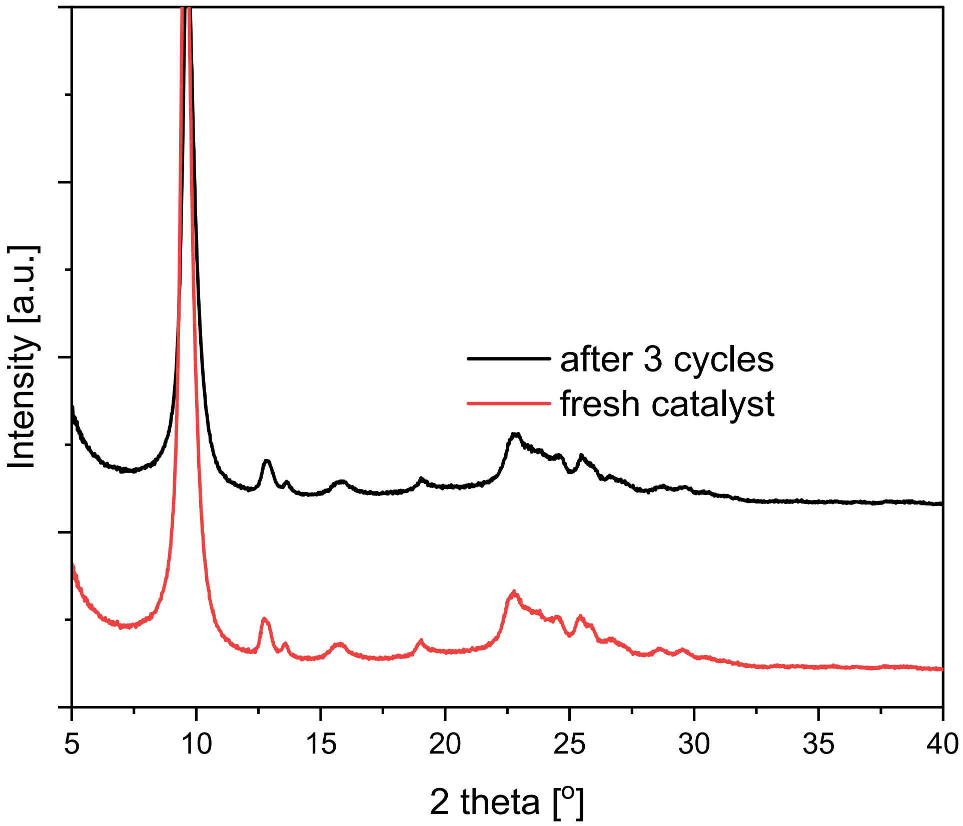

Fig. S1. X-ray diffractograms of fresh and used (after 3 catalytic runs) ITQ-6_30 sample. D2 PHASER powder diffractometer (Bruker, Billerica, MA, USA), diffractograms were taken in the 2θ range of 2-40 with a step of 0.02° and a counting time of 1 s per step.
